# Exosome–Liposome Hybrid Nanoparticles Deliver CRISPR/Cas9 System in MSCs

**DOI:** 10.1002/advs.201700611

**Published:** 2018-01-30

**Authors:** Yao Lin, Jiahua Wu, Weihuai Gu, Yulei Huang, Zhongchun Tong, Lijia Huang, Jiali Tan

**Affiliations:** ^1^ Department of Orthodontics Guanghua School of Stomatology Hospital of Stomatology Sun Yat‐sen University Guangdong Provincial Key Laboratory of Stomatology Guangzhou 510055 P. R. China; ^2^ Guangdong Provincial Key Laboratory of Stomatology Guanghua School of Stomatology Hospital of Stomatology Sun Yat‐sen University Guangzhou 510055 P. R. China

**Keywords:** CRISPR/Cas9 system, exosomes, hybrid nanoparticles, targeted delivery

## Abstract

Targeted delivery of clustered regularly interspaced short palindromic repeats (CRISPR)/CRISPR‐associated protein 9 (Cas9) system to the receptor cells is essential for in vivo gene editing. Exosomes are intensively studied as a promising targeted drug delivery carrier recently, while limited by their low efficiency in encapsulating of large nucleic acids. Here, a kind of hybrid exosomes with liposomes is developed via simple incubation. Different from the original exosomes, the resultant hybrid nanoparticles efficiently encapsulate large plasmids, including the CRISPR–Cas9 expression vectors, similarly as the liposomes. Moreover, the resultant hybrid nanoparticles can be endocytosed by and express the encapsulated genes in the mesenchymal stem cells (MSCs), which cannot be transfected by the liposome alone. Taken together, the exosome–liposome hybrid nanoparticles can deliver CRISPR–Cas9 system in MSCs and thus be promising in in vivo gene manipulation.

## 1 Introduction

Gene therapy is considered as a promising and radical treatment for diseases like cancers and inherited disorders.^1^ Since its innovation, the clustered regularly interspaced short palindromic repeats (CRISPR)/CRISPR‐associated protein 9 (Cas9) system has been recognized as the most promising gene‐editing and gene‐regulation technique.^2^ The CRISPR/Cas9 system is an adaptive immunological response found in archaea and bacteria preventing invasion like viruses and plasmids.^3^ The engineered CRISPR/Cas system works as a Cas9 nuclease‐single guide RNA (sgRNA) complex. The sgRNA recognizes the complementary 20‐nucleotide genomic sequence, and Cas9 nuclease generates double‐strand DNA breaks three bases upper stream of the protospacer adjacent motif of the target gene, resulting in gene deletion, insertion, and mutation by error‐prone nonhomologous end‐joining or precise homology‐directed repair.[[qv: 2a,4]] Besides, CRISPR/Cas9 system can also be engineered to regulate gene expression via fusing the gene regulator to the dead Cas9 (dCas9), in which the two catalytic domains of Cas9, RuvC and HNH, are inactivated and thus have no cleavage activity.^5^

Although CRISPR/Cas9 system emerges as a promising strategy for gene therapy, one major obstacle remains as there is no safe and efficient delivery method for CRISPR/Cas9 system in vivo. Currently, CRISPR/Cas9 system is delivered in vivo mostly via viral vectors, including lentiviral vectors, adenoviral vectors, and adeno‐associated virus (AAV) vectors.[[qv: 5b,6]] However, the possible cytotoxicity, immunogenic response, long‐term expression, and off‐target effects of viral vectors, continue to be the clinical application concern.^7^ Also, the nonviral delivery methods, such as electroporation,[[qv: 7a]] microinjection,^8^ lipid and lipid‐like nanoparticles delivery,^9^ and ribonucleoprotein delivery^10^ are reported, which are also limited by their stability, accessibility, safety, or efficiency.

Exosomes are nanoscale membrane vesicles with a diameter range of 30–100 nm, which are secreted by almost all kinds of cells and stably exist in virtually all kinds of bodily fluids.^11^ They can transmit a variety of signaling molecules, including nucleic acids mainly mRNA and microRNA, functional proteins, and lipids.^12^ Owing to the small size of exosomes, they can escape from the rapid phagocytosis by mononuclear phagocytes, steadily carry and deliver drugs in circulation, and pass through vascular endothelium to target cells.^13^ Exosomes can also cross the stringent biological barriers such as the blood–brain barrier and the placental barrier.^14^ It also has been reported that exosomes exhibit good tissue‐ or cell‐targeting owing to their particular surface proteins like tetraspanin.^15^ In addition, researchers can get better targetability via engineering surface molecules of exosomes. For instance, lamp2b fused with the neuron‐specific rabies viral glycoprotein (RVG) peptide can target neurons.^14^a All of these characteristics make exosome a promising in vivo drug delivery carrier.^14^, ^16^ However, due to the small size of exosomes, it is difficult to encapsulate large nucleic acids into exosomes. The current reports about exosomes as drug delivery vehicles are mostly related to small nucleic acids like miRNAs and siRNAs or low molecular medicines, which are much lower than the Cas9 expressing plasmids with the minimal size of 5–6 kb.^14^, ^17^ Therefore, it is urgent to find a suitable method to encapsulate the CRISPR/Cas9 system into exosomes.

In this study, we successfully encapsulated large nucleic acids, including the CRISPR/Cas9 expression vectors into hybrid exosomes, which were produced via incubating the original exosomes with liposomes. Besides, the loaded hybrid exosomes could be endocytosed by mesenchymal stem cells (MSCs) and released cargos inside. Finally, the hybrid exosomes delivery CRISPR/Cas9 system was validated to be expressed in MSCs and regulate the target gene expression, providing us a new strategy to deliver CRISPR–Cas9 system in MSCs for gene manipulation.

## 2 Results and Discussion

### 2.1 Identification and Characterization of HEK293FT Cell‐Derived Exosomes

**Figure 1:**
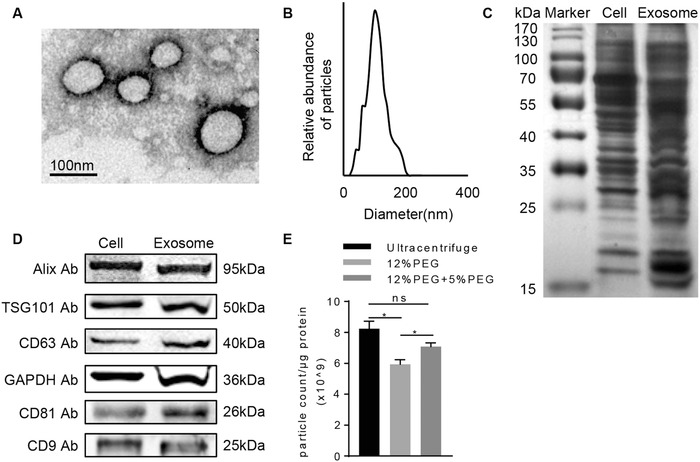
Characterization of HEK293FT cell‐derived exosomes. A) Representative electron microscopy image of the exosomes isolated from HEK293FT cells. Scale bar, 100 nm. B) Size distribution of HEK293FT cell derived exosomes determined by dynamic light scattering. Data represent 20 measurements of four biological samples. C) The protein profile of HEK293FT cells and the exosomes secreted by HEK293FT cells were analyzed by silver blotting. Representative image of three different experiments. D) The protein levels of Alix, TSG101, CD63, CD81, CD9, and GAPDH in HEK293FT cell lysates and the exosomes secreted by HEK293FT cells were analyzed by western blot. Data presented were representative of three different experiments. E) Particle‐to‐protein ratio of exosomes isolated by ultracentrifugation, 12% PEG 6000 precipitation and 12% precipitation + 5% PEG 6000 reprecipitation. Data were expressed as mean ± standard deviation (SD) of three different experiments. *p < 0.05.

HEK293FT cell‐derived exosomes were isolated from the culture supernatants via polyethylene glycol (PEG) 6000. We here chose HEK293FT cells as the source of exosomes for the following reasons. It has been reported that HEK293FT cells were productive in exosome biogenesis and secretion. And as the most available tool cells, HEK293FT cells were easy for gene transfection. Electron microscopy analysis confirmed the round‐shaped morphology of exosomes with visible lipid layer (**Figure** **1**A). Particle size distribution analysis further revealed that the exosomes ranged from 30 to 100 nm in diameter (Figure 1B and Figure S1 (Supporting Information)). To further confirm the identity of exosomes, we extracted the proteins from HEK293FT cells lysates and exosomes secreted by HEK293FT cells. The image of silver staining showed that exosomes contained various proteins of different molecular sizes (Figure 1C). Moreover, the western blot result confirmed the presence of exosomal marker proteins, such as Alix, TSG101, CD63, CD81, and CD9 (Figure 1D).

Notably, we tried to encapsulate large DNA into HEK293FT cell‐derived exosomes and transfer the exosomes to MSCs. In addition, enhanced green fluorescent protein (EGFP) and CRISPR/Cas9 expression were driven by constative promoters, which are confirmed to work very well in both murine and human cells.

Considering the possibility of co‐isolated contaminants via PEG 6000, we assessed the purity of the isolated exosomes via the particle‐to‐protein ratio. As expected, there was a slight protein contamination in the PEG 6000 precipitation method, as repeated wash–centrifugation would reduce the protein concentration per exosome. However, the protein contamination was comparable to that in the ultracentrifuge method (Figure 1E). Since the following fusion process was achieved in the solution, the dissolved protein might be not a big concern in this study. However, it should be settled for the future translational study.

### 2.2 Construction of Hybrid Exosomes for Large Plasmid Encapsulation

Exosomes have been applied as drug delivery vehicles and transferred various cargos. Methods published for encapsulating drugs in exosomes include electroporation, incubation, or transfection of donor cells.^14^, ^18^ It was also reported that catalase loaded into exosomes via sonication and extrusion, or permeabilization with saponin results in higher loading efficiency than simple incubation. And the cellular uptake efficiency of sonicated exosomes was increased.^19^ Most of the studies show the ability of exosomes to encapsulate and transfer small nucleic acids like miRNAs, siRNAs to target cells and organs and exhibit therapeutic effects.^14^, ^20^ Other cargos, proteins (vesicular stomatitis virus G protein),[[qv: 18b]] and small molecules (doxorubicin, curcumin, cisplatin, and so on)[[qv: 18a,21]] are also reported. However, due to the small size of exosomes, exosomes have rarely been reported as exogenous DNA carriers. It was recently indicated that plasmid DNA could be loaded into extracellular vesicles via transfection of donor cells, with only the plasmid DNA delivered by microvesicles resulting in functional protein expression but not exosomes.^22^ Besides, it was demonstrated that linear DNA less than 1000 bp in length could be efficiently loaded in extracellular vesicles via electroporation compared to larger linear DNAs and plasmid DNAs, and the DNA loading capacity of microvesicles was much higher than exosomes.^23^

**Figure 2:**
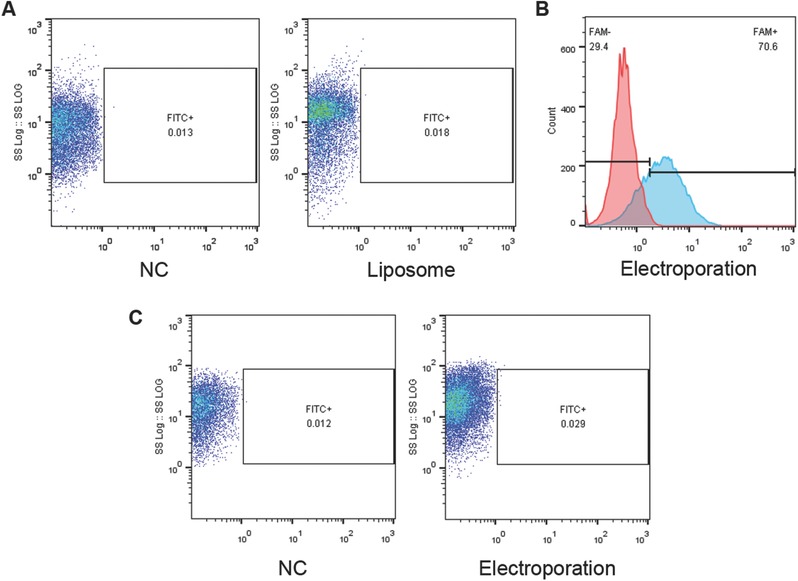
Liposome or exosome alone fails to deliver plasmids into MSC. A) FACS analysis of green fluorescence protein expression in MSCs with transfection of control or pEGFP‐C1 plasmids via liposomes. B) FACS analysis of FAM level in MSCs with or without incubation of exosomes electroporated with control or FAM conjugated small RNA. C) FACS analysis of green fluorescence protein expression in MSCs with incubation of exosomes electroporated with control or pEGFP‐C1 plasmids.

In this study, we attempted to encapsulate large size plasmid DNA into exosomes and transfer to MSCs. Consistent with the previous notion that MSCs were resistant to Lipofectamine 2000 mediated transfection, fluorescence activated cell sorter (FACS) results confirmed that Lipofectamine 2000 failed to transfect the pEGFP‐C1 plasmids into MSCs (**Figure** **2**A). Moreover, electroporation loaded exosomes could transfect small RNA efficiently into MSCs (Figure 2B), while failed to transfect the pEGFP‐C1 plasmids (Figure 2C), possibly due to the failure of encapsulating the plasmids via electroporation. Together, these data indicate that species difference between HEK293FT cells and murine MSCs is not the hurdle for exosome transfection, while the inability to encapsulate the large DNA into the exosome is the problem.

**Figure 3:**
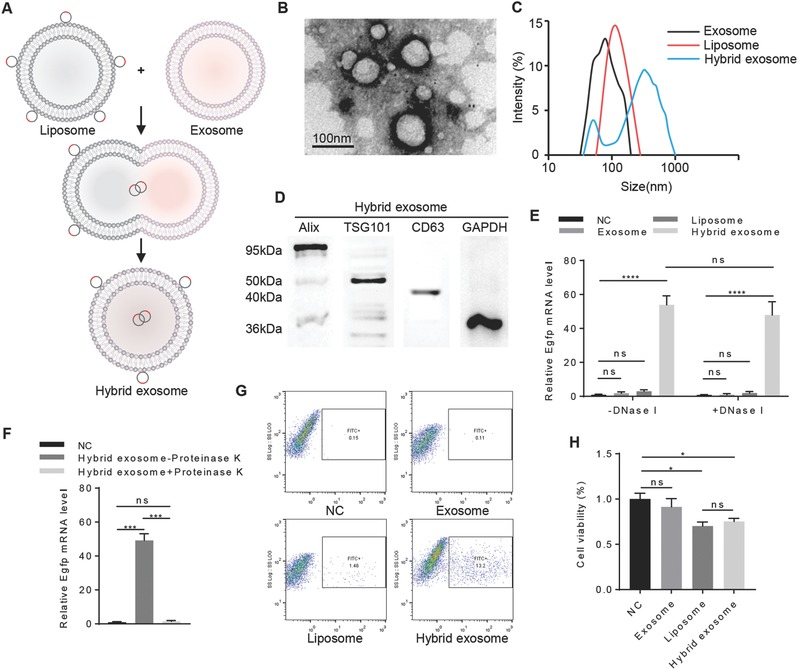
Assessment of hybrid exosomes encapsulating large plasmids. A) Illustration of the procedure to produce hybrid exosomes. The isolated exosomes were incubated with liposomes for 12 h at 37 °C to induce the fusion. B) Representative electron microscopy image of exosomes after incubation with liposomes for 12 h at 37 °C. Scale bar, 100 nm. C) Size distribution of exosomes, liposomes, and hybrid exosomes determined by dynamic light scattering. Data represent 20 measurements of four biological samples. D) Western blot analysis of the protein levels of Alix, TSG101, CD63, and GAPDH in hybrid exosomes. Data presented were representative of three different experiments. E) qRT‐PCR analysis of EGFP mRNA level in the MSCs with or without DNase treatment prior to incubation with pEGFP‐C1 plasmid‐only (NC), exosomes+pEGFP‐C1 plasmid (exosome), Lipofectamine2000+pEGFP‐C1 plasmid (liposome) and hybrid exosomes+pEGFP‐C1 plasmid (hybrid exosome). Data were expressed as mean ± SEM of three different experiments. *p < 0.05. F) qRT‐PCR analysis of EGFP mRNA level in the MSCs with incubation of pEGFP‐C1 plasmid‐only (NC) or hybrid exosome+pEGFP‐C1 (Hybrid exosome) with and without proteinase K treatment. Data were expressed as mean ± standard error of the mean (SEM) of three different experiments. *p < 0.05. G) FACS analysis of green fluorescence protein expression of MSCs with incubation of the nanoparticle same as (E). H) Cell viability of MSCs with incubation of the nanoparticles same as (E). Data were expressed as mean ± SD of three different experiments. *p < 0.05.

Then, we tried to produce hybrid exosomes for loading large plasmids. Exosomes were incubated with the mixture of liposomes and pEGFP‐C1 plasmids for 12 h at 37 °C (**Figure** **3**A). The incubation procedure resulted in the fusion of exosomes with liposomes (Figure 3B), and thus encapsulation of the plasmids. Among the conditions we tried, 12 h incubation was the best time, balancing the fusion efficiency and the stability, as determined by the best transfection efficiency and forced gene expression (Figure S2, Supporting Information). And, we also found that when the volume ratio of exosomes (about 10^9^ in 100 µL) to Lipofectamine 2000 was 2, the EGFP mRNA level in MSCs was higher after incubation (Figure S3, Supporting Information). As is reported before, the fusion between liposomes and exosomes might be due to the lipid structure of these two nanoparticles. To further confirm the generation of hybrid exosomes, we analyzed the size distribution of exosomes, liposomes, and hybrid nanoparticles, which suggested the successful fusion of exosomes with liposomes (Figure 3C). And western blot analysis of exosomal markers (Figure 3D), further confirming the exosomal composition of the hybrid nanoparticles.

In order to determine whether the DNA was truly encapsulated in the exosome–liposome hybrids or it is present extracellularly or attached to the surface of those nanoparticles, MSCs were incubated with hybrid nanoparticles with or without DNase treatment prior to incubation. Quantitative real‐time PCR (qRT‐PCR) analysis of EGFP mRNA level in the MSCs indicated that the DNA was mostly encapsulated in the exosome–liposome hybrids and efficiently transfected into the MSCs (Figure 3E). In addition, qRT‐PCR analysis showed that proteinase K treated hybrid nanoparticles failed to deliver the genes efficiently in the MSCs (Figure 3F), which indicated that the hybrid nanoparticles could be efficiently taken up by MSCs due to the vesicle proteins retaining on the surface. The FACS results also revealed that large plasmids could be delivered into MSCs only with hybrid exosomes (Figure 3G).

Considering the possible toxic effects of liposomes, we also analyzed the cell viability after incubating with each vehicle or hybrid nanoparticles. Similar toxic effects of the liposomes (Lipofectamine) and hybrid exosomes have been observed, while exosome only had no obvious toxic effects (Figure 3H). Therefore, further modification of the liposome components should be better done before its clinical application.

### 2.3 Construction and Examination of CRISPR/Cas9 Based Runx2 Gene Expression Regulation System

CRISPR/Cas9 system has been highly valued these years compared to the conventional nuclease tools, zinc‐finger nucleases (ZFNs), and transcription activator‐like effector nucleases (TALENs). Although they all work similarly for the requirement of a DNA‐binding molecule and an endonuclease domain, CRISPR/Cas9 system possesses some advantages. First of all, the construction and optimization of custom ZFNs and TALENs are complicated, expensive, and time‐consuming. However, CRISPR/Cas9 system just needs synthesized 20‐base‐long sequences to get specificities instead of demanding engineered proteins.^24^ Besides, CRISPR/Cas9 system has been reported to get higher sequence specificity and targeting efficiency than ZFNs and TALENs.^24^, ^25^ Finally, CRISPR/Cas9 system can target multiple sites and induce multiple effects simultaneously, which ZFNs and TALENs lack.[[qv: 2b,26]] Therefore, the emerging of CRISPR/Cas9 system makes a breakthrough in gene therapy. The conventional method for CRISPR–Cas9 system delivery is viral vectors. However, due to the possible cytotoxicity, immunogenic response, long‐term expression, and off‐target effects, the applications of viral vectors are limited. Currently, there are no efficient and safe non‐viral delivery carriers for CRISPR–Cas9 system. The proposed hybrid exosomes provided here at least could be a candidate for future in vitro and in vivo studies.

**Figure 4:**
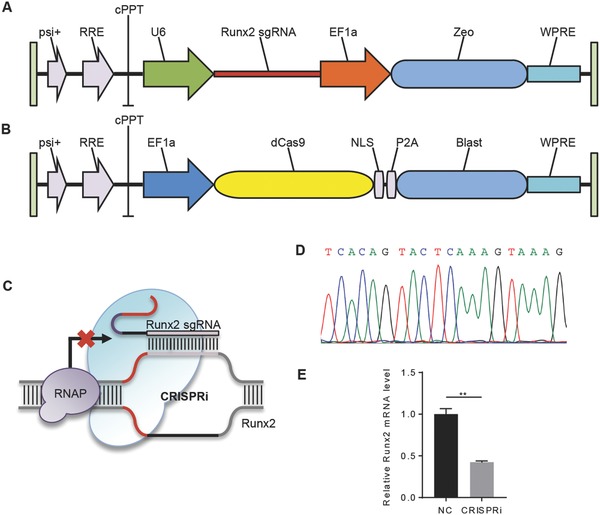
Construction of CRISPR/dCas9 based Runx2 expression intervention system. A) Diagram of Runx2 sgRNA expressing lentiviral vector. B) Diagram of dCas9 expressing lentiviral vector. C) Illustration of CRISPR/dCas9 based Runx2 gene expression intervention system. D) Sequencing results of sgRNA construct targeting Runx2 gene. E) qRT‐PCR analysis of Runx2 mRNA level in MSCs infected with the control or Runx2 guided CRISPR/dCas9 system. Data were expressed as mean ± SEM of three different experiments. *p < 0.05.

To test whether the hybrid exosome could be used to deliver CRISPR/Cas9 system into MSCs, we constructed a CRISPR interference system targeting mRunx2 gene and a CRISPR cleavage system targeting hCTNNB1 gene. The CRISPR interference system works as a sgRNA–dCas9 complex to repress the gene expression. Briefly, the sgRNA recognizes the complementary sequence of Runx2 gene, and dCas9 physically blocks RNA polymerase binding during transcription elongation. The CRISPR interference system consisted of two vectors, lenti sgRNA‐zeo and lenti dCas9‐Blast. sgRNAs were designed using the online CRISPR Design Tool and ligated into sgRNA expressing vectors using BsmBI enzyme and confirmed by DNA sequencing (**Figure** **4**A–D). As expected, the resultant lentivirus infection of MSCs successfully reduced Runx2 expression via qRT‐PCR analysis (Figure 4E).

**Figure 5:**
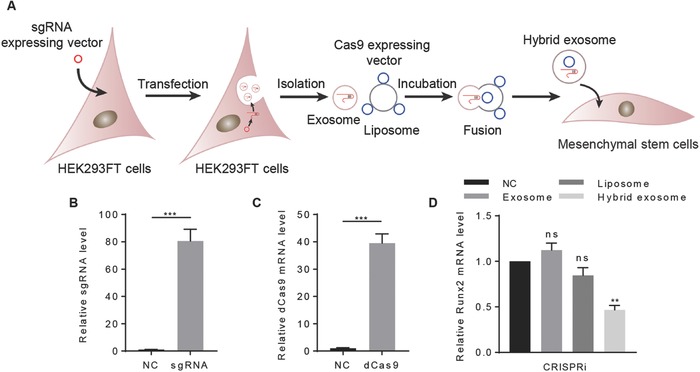
The hybrid exosomes successfully deliver CRISPR/dCas9 interference system. A) Illustration of procedure how the hybrid exosomes deliver the CRISPR/dCas9 interference system. B) qRT‐PCR analysis of sgRNA level in MSCs with control or Runx2 gRNA expressing hybrid exosomes. Control empty vector served as NC. Data were expressed as mean ± SEM of three different experiments. *p < 0.05. C) qRT‐PCR analysis of dCas9 mRNA level in MSCs with control or dCas9 expressing plasmids loaded hybrid exosomes. Control empty vector served as NC. Data were expressed as mean ± SEM of three different experiments. *p < 0.05. D) Runx2 mRNA level in MSCs with incubation of Runx2 guided CRISPR/dCas9 system‐only (NC), exosomes+ Runx2 guided CRISPR/dCas9 system (exosome), liposomes+ Runx2 guided CRISPR/dCas9 system (liposome), and hybrid exosomes+Runx2 guided CRISPR/dCas9 system (hybrid exosome). Data were expressed as mean ± SEM of three different experiments. *p < 0.05.

Next, the hybrid exosome strategy was used to deliver CRISPR/Cas9 system. We incubated exosomes derived from sgRNA expressing cells with the mixture of liposomes and dCas9 expressing vector for 12 h at 37 °C and then added to the culture medium of murine MSCs (**Figure** **5**A). Compared with the control group, the hybrid exosomes substantially increased both sgRNA and dCas9 mRNA expression (Figure 5B,C). Consistently, Runx2 expression was significantly decreased upon hybrid exosome mediated CRISPR/dCas9 targeted Runx2 delivery (Figure 5D), which indicated that CRISPR system could be loaded into the hybrid exosomes and delivered to recipient cells for gene engineering.

Since the endogenous RNA and protein could be encapsulated into the exosomes, we thus tested whether the isolated exosomes derived from the transfected cells had functional sgRNA and CRISPR/Cas9. qRT‐PCR results showed that both dCas9 and sgRNA were included in the exosomes (Figure S4A,B, Supporting Information). Although Cas9 mRNA appears in the exosome, the full‐length Cas9 mRNA was much less (Figure S4C, Supporting Information). Moreover, we also found that dCas9 proteins could be detected in the exosomes via western blot (Figure S4D, Supporting Information). However, isolated exosomes derived from the transfected cells and added to the exosomes had minimal effects on Runx2 expression when those exosomes were used to treat bone MSCs (Figure S4E, Supporting Information). The results implicated that dCas9 protein loaded in exosomes was not functional, which might be due to inactivation of the enzyme caused by structure conformation change. Alternatively, it might be explained by the inefficacy to encapsulate the protein via this strategy.

### 2.4 Hybrid Exosomes Successfully Deliver CRISPR/Cas9 Cleavage System into MSC

**Figure 6:**
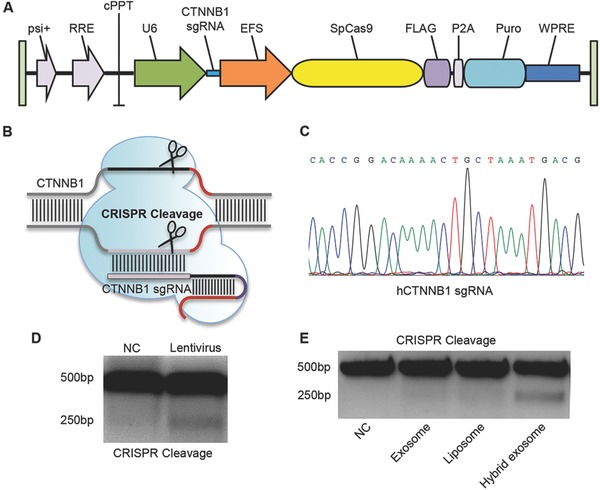
Construction of CRISPR/Cas9 based CTNNB1 cleavage system and its efficiency in editing gene via virus and hybrid exosome. A) Diagram of CTNNB1 sgRNA and Cas9 expressing lentiviral vector. B) Illustration of CRISPR/Cas9 based CTNNB1 cleavage system. C) Sequencing results of sgRNA construct targeting CTNNB1 gene. D) T7E1 assay results of MSCs infected with the control or CTNNB1 guided CRISPR/Cas9 system. E) T7E1 assay results of MSCs with incubation of CTNNB1 guided CRISPR/Cas9 system‐only (NC), exosomes+CTNNB1 guided CRISPR/Cas9 system (exosome), liposomes+CTNNB1 guided CRISPR/Cas9 system (liposome), and hybrid exosomes+CTNNB1 guided CRISPR/Cas9 system (hybrid exosome). Images are the representative of three different experiments.

The widely used CRISPR/Cas9 cleavage system works as a sgRNA–Cas9 complex for gene editing. To this end, one sgRNA recognized hCTNNB1 gene was cloned and used together with the lentiCRISPRv2 (**Figure** **6**A–C). As expected, the Cas9 nuclease generated double‐strand DNA breaks of the hCTNNB1 gene, as shown by the T7 endo I assay (Figure 6D).

Similarly, we incubated human MSCs with lentiCRISPRv2 alone or pre‐incubated with exosome, lipofectamine, or the hybrid exosomes. T7 endo I assay detected the mismatch of hCTNNB1 gene only in the hybrid exosome group, which further indicated that the CRISPR cleavage system could be also loaded into the hybrid exosomes (Figure 6E) and work in the recipient cells.

## 3 Conclusion

In summary, we produced hybrid exosomes via simple incubating exosomes with liposomes and successfully delivered CRISPR–Cas9 system in MSCs via hybrid exosomes for the first time. The application of CRISPR/Cas9 system has been a significant breakthrough in gene therapy remaining intractable over decades. Researchers have demonstrated the possibility of using CRISPR/Cas9 system to cure various genetic diseases such as cancers and inherited disorders via repairing, deleting, or silencing certain genetic mutations relating to the diseases in vivo or even to clinical trials in the future.^27^ For example, one study revealed that CRISPR/Cas9 system could treat Duchenne muscular dystrophy via deleting exon 23 of the dystrophin gene and improving muscle function in a mouse model.[[qv: 27c]] However, there still exist some challenges when it comes to applying in clinical. One major difficulty is the lack of more efficient delivery and safer delivery system. Although virus vectors like AAV have shown great efficacy in CRISPR/Cas9 system delivery, people still concern about the immunogenic response and long‐term expression of virus vectors, which prevent the wider applications of virus vectors in clinical. In this study, we attempted different approaches to encapsulate CRISPR–Cas9 system into exosomes and found out that the proposed hybrid exosome via incubating with liposomes could be a new strategy for drug encapsulating and delivering CRISPR–Cas9 system in vivo or in transfection resistant cells in vitro.

## 4 Experimental Section

*Cell Culture*: HEK293FT cells and human MSCs were cultured in high‐glucose Dulbecco's modified Eagle medium (DMEM) containing 10% fetal bovine serum (FBS) and 1% penicillin/streptomycin under 37 °C and 5% CO_2_. The cells were passaged at a ratio of 1:8 at 90% confluency.

All animal experiments were carried out according to the Sun Yat‐sen University guidelines and approved by the Institutional Animal Care and Use Committee of the Sun Yat‐sen University. MSCs were isolated from the mouse bone marrow. Briefly, mice (C57BL/6, six weeks old) were killed by cervical dislocation and soaked in 75% alcohol for 5 min. Then, femur and tibia were separated with sterile operating procedures. The bone marrow was flushed by a syringe into a plate with complete media. MSCs were plated in a 25 cm^2^ flask and incubated in DMEM containing 10% FBS and 1% penicillin/streptomycin at 37 °C and 5% CO_2_ without disturbing. After 72 h, nonadherent cells were removed by changing the medium to complete fresh medium. Cells were split at a ratio of 1:3 until ≈80% confluency was reached.

*Exosome Isolation*: HEK293FT cells cultured with full medium at 80–90% confluency were replaced with fresh medium without FBS. After 48 h culture, the cell medium was harvested and centrifuged at 500*g* for 30 min and 12 000*g* for an additional 30 min sequentially to remove cell debris. Exosomes were isolated from the cell medium using PEG 6000 with the final concentration of 12% in 500 × 10^−3^ m NaCl solution. The mixture was then incubated at 4 °C overnight and centrifuged at 10 000*g* for 1 h. Exosome pellets were resuspended in phosphate buffer saline (PBS) or DMEM and stored at −20 °C.

*Transmission Electron Microscopy*: Isolated exosomes were added on a copper grid and were stained with phosphotungstic acid 10 min later. The dried grids were examined using the transmission electron microscope.

*Dynamic Light Scattering (DLS)*: Size distribution of exosomes, liposomes, and hybrid nanoparticles was measured by DLS as described before.

*Western Blotting*: HEK293FT cells or exosomes derived from HEK293FT cells were lysed with radio‐immunoprecipitation assay buffer (RIPA buffer) and quantified with the bicinchoninic acid assay (BCA assay) 50 µg of sample proteins were separated via the sodium dodecyl sulfate polyacrylamide gel electrophoresis (SDS‐PAGE) gel electrophoresis and transferred to nitrocellulose filter membrane (NC membranes). Membranes were blocked with 5% skim milk at room temperature for 1 h, then washed three times with Tris‐buffered saline tween buffer (TBST) and incubated with anti‐mouse Alix (Santa Cruz Biotechnology), anti‐rabbit TSG101 (System Biosciences), anti‐rabbit CD9 (System Biosciences), anti‐rabbit CD81 (System Biosciences), anti‐mouse CD63 (Santa Cruz Biotechnology), anti‐rabbit glyceraldehyde‐3‐phosphate dehydrogenase (GAPDH) antibodies diluting in Tris‐buffered saline buffer at 4 °C overnight. Anti‐rabbit GAPDH antibody was used as a control protein. Membranes were washed in TBST and incubated with anti‐rabbit immunoglobulin G (IgG) horseradish peroxidase (HRP)‐linked antibody (Cell Signaling Technology) or anti‐mouse IgG HRP‐linked antibody (Cell Signaling Technology) at room temperature for 1 h followed by TBST washing. The expected bands were detected by ECL Prime Western Blotting System (GE Healthcare) and visualized on a Tanon 5500 chemiluminescent imaging system. Quantities of Cas9 in transfected cells and exosomes from the transfected cells were also detected in the same way with the anti‐Cas9 antibody (Cell Signaling Technology).

*Silver Staining*: Sample proteins from cells and exosomes were separated on a 10% SDS‐PAGE gel and visualized by a Fast Silver Stain Kit (Tiangen). First, the gel was fixed for 20 min in 50% ethanol:10% acetic acid solution. Then, the gel was washed for 10 min in 30% ethanol, followed by 10 min wash in ultrapure water. Then, the gel was sensitized for 2 min in sensitizer, followed by 1 min washes with water twice. Finally, the gel was stained for 10 min and washed twice with water. The gel was developed for 3–10 min and stopped until bands appeared.

*Plasmid Construction*: sgRNAs targeting mRunx2 gene and hCTNNB1 gene were designed using the online CRISPR Design Tool (http://tools.genome-engineering.org). The synthesized paired oligos were diluted in sterile water and annealed in a thermal cycler. The annealed oligos were then cloned into the lenti sgRNA backbone after BsmBI digestion.

*Loading of Exosomes*: Exosomes were loaded with cargos via three different strategies.

For electroporation, exosomes and carboxyfluorescein (FAM) were mixed in 400 µL electroporation buffer and electroporated at 350 V and 150 µF in a 2 mm cuvette using a Gene Pulser II Electroporator. The mixture was incubated at 4 °C for 30 min for recovery before added into the MSCs. The FAM level was analyzed by flow cytometry after 6 h.

Transfection of cells was also used for exosomes loading. Briefly, HEK293FT cells were transfected with CRISPR/Cas9 vectors using Lipofectamine 2000. 6 h after transfection, the cell medium was replaced with fresh media without FBS. After 48 h culture, the exosomes were isolated from the harvested cell media with the same purification reagents mentioned above.

Hybrid exosomes were produced by incubating with liposome. Lipofectamine 2000 and indicated plasmids were diluted in DMEM, respectively, then mixed and incubated at room temperature for 5 min. Then, the plasmid–liposome complex was added to exosomes and incubated at 37 °C for 12 h.

*Transfection*: MSCs were plated in a six‐well plate and transfected with pEGFP‐C1 plasmids when 80% confluency was reached. pEGFP‐C1 plasmids and Lipofectamine 2000 diluted in serum‐free DMEM were mixed with 5 min incubation. Then, the complex was added to the MSC culture, and the medium was replaced with fresh complete DMEM after 6 h incubation. The transfected cells were analyzed after 24 h. sgRNA expressing vector and dCas9 expressing vector were transfected into MSCs or HEK293FT cells in the same way.

*Lentivirus Packaging and Infection*: HEK293FT cells were seeded in a six‐well plate and transfected with the expression vectors (lenti sgRNA or lenti dCas9‐Blast), pMD2.G (the envelope vector), and psPAX2 (the packaging vector) at a 5:5:1 ratio when 80% confluency was reached. The medium was replaced with 2 mL fresh complete DMEM at 12 h, which was then collected at 72 h. The virus‐containing medium was then filtered with 0.45 µm filter and stored at −80 °C.

For infection, MSCs were seeded in a six‐well plate and infected when 70% confluency was reached. Briefly, 1 mL virus‐containing medium, 1 mL complete medium, and polybrene with the final concentration of 8 µg µL^−1^ were mixed and added to the cells, which were then replaced with fresh medium at 12 h. Infection efficiency and effects were analyzed at 72 h or later.

*Flow Cytometry*: Cells transfected with pEGFP‐C1 plasmids via liposomes or incubated with hybrid exosomes loading pEGFP‐C1 plasmids, were washed with PBS, digested with trypsin enzyme, and resuspended in PBS. The cells were then analyzed by a flow cytometer at an excitation wavelength of 480 nm and an emission wavelength of 520 nm.

*PCR*: Total RNA was harvested from cells and exosomes with and without transfection using TRIzol reagent. A total of 2 µg RNA per reaction was used to generate cDNA via Transcriptor First Strand cDNA Synthesis Kit (Roche). qRT‐PCR experiments were carried on the CFX96 System (BIO‐RAD) in 20 µL reactions, with 500 × 10^−9^ m of each primer, 50 ng cDNA, and 10 µL 2 × SYBR Green I Master Mix. For semiquantitative PCR analysis, the reaction was run in a PCR cycler: 4 min, 95 °C; 30 s, 95 °C; 30 s, 58 °C; 4 min, 72 °C; go back to step2 by 30 cycles; 7 min, 72 °C; hold at 4 °C. The PCR product was run on a 1% (wt/vol) agarose gel. PCR primers used this experiment are listed in Table S1 (Supporting Information).

*T7 Endonuclease 1 (T7E1) Assay*: Genomic DNA was extracted for confirmation of the indels or mutations using PCR amplification with the primers listed in Table S1 (Supporting Information). T7E1 assay was performed as detailed before.

*Statistical Analysis*: Data analyses were performed with *t*‐test or analysis using statistical package SPSS 24. *p* < 0.05 was considered statistically significant.

## Conflict of Interest

The authors declare no conflict of interest.

## Supporting information

Supplementary
