# Supplementary material for: Exosome–Liposome Hybrid Nanoparticles Deliver CRISPR/Cas9 System in MSCs
Source: Adv Sci (Weinh). 2018 Jan 30;5(4):1700611. doi: 10.1002/advs.201700611 (PMC5908366; doi:10.1002/advs.201700611)
Supplement: Supplementary file 1 — Supplementary [file ADVS-5-1700611-s001.pdf]

Copyright WILEY-VCH Verlag GmbH & Co. KGaA, 69469 Weinheim, Germany, 2017.

ADVANCED SCIENCE
Open Access

Supporting Information

for *Adv. Sci.,* DOI: 10.1002/advs.201700611

Exosome–Liposome Hybrid Nanoparticles Deliver CRISPR/Cas9 System in MSCs

*Yao Lin, Jiahua Wu, Weihuai Gu, Yulei Huang, Zhongchun Tong, Lijia Huang, and Jiali Tan**

Copyright WILEY-VCH Verlag GmbH & Co. KGaA, 69469 Weinheim, Germany, 2016.

Supporting Information

Title: Exosome-Liposome Hybrid Nanoparticles Deliver CRISPR/Cas9 System in MSCs

*Yao Lin, Jiahua Wu, Weihuai Gu, Yulei Huang, Zhongchun Tong, Lijia Huang and Jiali Tan**

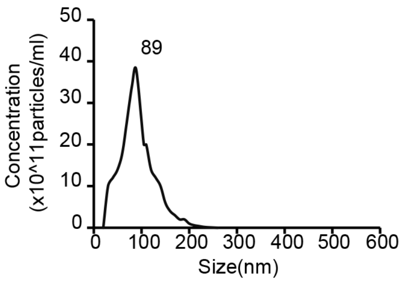

Figure S1. Size distribution of HEK293FT cell derived exosomes determined by Nanoparticle Tracking Analysis.

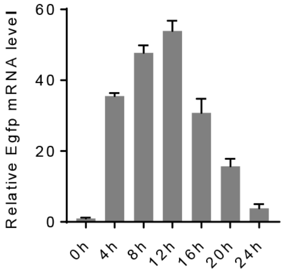

Figure S2. qRT-PCR analysis of EGFP mRNA level in the MSCs treated with hybrid exosomes encapsulating pEGFP-C1 plasmid manipulated by incubation for 0h, 4h, 8h, 12h, 16h, 20h and 24h.

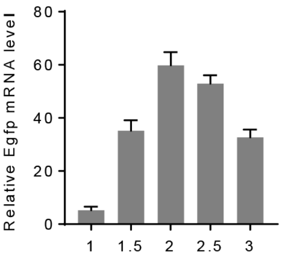

Figure S3. qRT-PCR analysis of EGFP mRNA level in the MSCs treated with the hybrid exosomes manipulated by incubating indicated ratio of exosomes (10^9 in 100 µL)to liposomes.

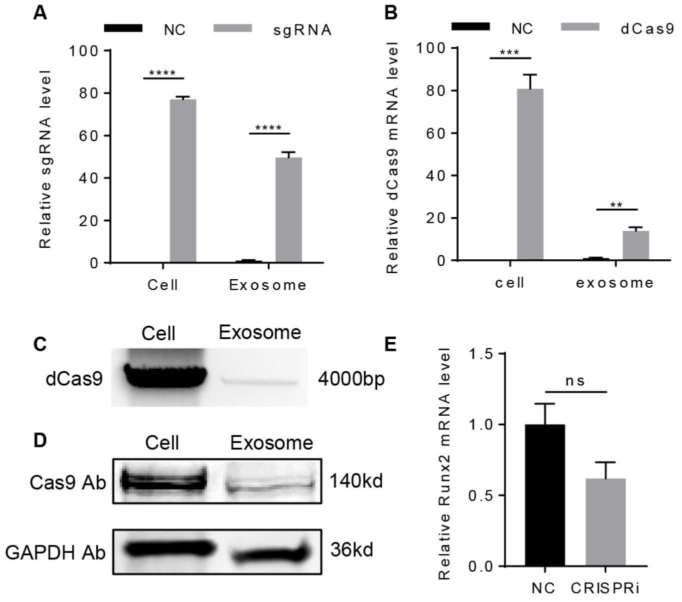

Figure S4. Transfection in cells fails to encapsulate CRISPR/dCas9 into exosomes. (A) qRT-PCR analysis of cellular and exosomal sgRNA levels in HEK293FT cells and the derivative exosomes. Cells were transfected with control or sgRNA expressing lentiviral vector. Data were expressed as mean±SEM of three different experiments. *p<0.05. (B) qRT-PCR analysis of cellular and exosomal dCas9 mRNA levels in HEK293FT cells and the derivative exosomes. Cells were transfected with control or dCas9 expressing lentiviral vector. Data were expressed as mean±SEM of three different experiments. *p<0.05. (C) Semi-quantitative PCR analysis of the expression of full length of dCas9 mRNA in HEK293FT cells and the derivative exosomes. Cells were transfected with dCas9 expressing lentiviral vector. Representative image of three different experiments. (D) The protein levels of Cas9 and GAPDH in HEK293FT cells and the derivative exosomes. Cells were transfected with dCas9 expressing lentiviral vector. GAPDH served as internal control. Representative image of three different experiments. (E) Runx2 mRNA level in mesenchymal stem cells with the exosomes derived from HEK293FT cells transfected with control or Runx2 sgRNA plus dCas9

expressing plasmids. Data were expressed as mean±SEM of three different experiments. *$p<0.05$.

Table S1. Sequences of the primers used in the study.

Primer	Sequence
mRunx2 sgRNA F	CACCGTCACAGTACTCAAAGTAAAG
mRunx2 sgRNA R	AAACCTTTACTTTGAGTACTGTGAC
hCTNNB1 sgRNA F	CACCGGACAAAACTGCTAAATGACG
hCTNNB1 sgRNA R	AAACCGTCATTTAGCAGTTTTGTCC
dCas9 PCR F	GGAAGCTTCCATGGACAAGAAGTACAGCATCGG
dCas9 PCR R	CCGGATCCTTAAGCGGCCGCCACCTTCCTCTT
hCTNNB1 PCR F	CTGAAAGTCAGAATGCAGTTTTGAG
hCTNNB1 PCR R	TGGTATTGGGTAGACATTCTGAAAC
eGFP qPCR F	CTCGTGACCACCCTGACCTAC
eGFP qPCR R	GTTCACCTTGATGCCGTTCTT
mRunx2 qPCR F	GGAGTGGACGAGGCAAGAGTTT
mRunx2 qPCR R	AGCTTCTGTCTGTGCCTTCTGG
dCas9 qPCR F	GTGGACGCTATCGTGCCT
dCas9 qPCR R	GCCGCCAGTAGTTCTTCAT
mRunx2 sgRNA qPCR F	AAAGATTGAGAAAGAGGGAGT
mRunx2 sgRNA qPCR R	GCCAAGTTGATAACGGACTA
mGAPDH qPCR F	GTGAAGGTCGGTGTGAAC
mGAPDH qPCR F	TGAGTGGAGTCATACTGGAA
